# Delivering COVID-19 vaccine trials at speed: the implementation of a phase IV UK multi-centre randomised controlled trial to determine safety and immunogenicity of COVID-19 vaccines co-administered with seasonal influenza vaccines (ComFluCOV)

**DOI:** 10.1186/s13063-023-07862-4

**Published:** 2024-01-11

**Authors:** Sarah Baos, Rachel Todd, Russell Thirard, Rosie Harris, Jana Kirwan, Katherine Joyce, David Hutton, Adam Finn, Madeleine Clout, Heike Cappel-Porter, Chris A Rogers, Rajeka Lazarus, Lucy Culliford

**Affiliations:** 1 Bristol Trials Centre, University of Bristol, Bristol, UK; 2 Bristol Vaccine Centre, Schools of Population Health Sciences and of Cellular and Molecular Medicine, University of Bristol, Bristol, UK; 3 University Hospitals Bristol and Weston NHS Foundation Trust, Bristol, UK

**Keywords:** COVID-19 vaccine, Influenza vaccine, Randomised controlled trial (RCT), Efficient delivery, Urgent Public Health (UPH)

## Abstract

**Background:**

In February 2021, the UK Department of Health and Social Care sought evidence on the safety and immunogenicity of COVID-19 and influenza vaccine co-administration to inform the 2021/2022 influenza vaccine policy. Co-administration could support vaccine uptake and reduce healthcare appointments. ComFluCOV was a randomised controlled trial designed to provide this evidence. This report outlines the methods used to deliver the trial in 6 months to answer an urgent public health question as part of the COVID-19 pandemic response.

**Methods:**

ComFluCOV was commissioned by the Department of Health and Social Care and was managed by the Bristol Trials Centre, a UK-registered clinical trials unit. It was classed as an Urgent Public Health trial which facilitated fast-track regulatory approvals. Trial materials and databases were developed using in-house templates and those used in other COVID-19 vaccine trials. Participants were recruited by advertising, and via a trial website. Electronic trial systems enabled daily review of participant data. Weekly virtual meetings were held with stakeholders and trial sites.

**Results:**

ComFluCOV was delivered within 6 months from inception to reporting, and trial milestones to inform the Department of Health and Social Care policy were met. Set-up was achieved within 1 month. Regulators provided expedited reviews, with feedback ahead of submission. Recruitment took place at 12 sites. Over 380 site staff were trained. Overall, 679 participants were recruited in two months. The final report to the Department of Health and Social Care was submitted in September 2021, following a preliminary safety report in May 2021. Trial results have been published.

**Conclusion:**

The rapid delivery of ComFluCOV was resource intensive. It was made possible in part due to a unique set of circumstances created by the pandemic situation including measures put in place to support urgent public health research and public support for COVID-19 vaccine research. Elements of the trial could be adopted to increase efficiency in ‘non-pandemic’ situations including working with a clinical trials unit to enable immediate mobilisation of a team of experienced researchers, greater sharing of resources between clinical trials units, use of electronic trial systems and virtual meetings.

**Trial registration:**

ISRCTN14391248, submitted on 17/03/2021. Registered on 30/03/2021.

## Background

Mass vaccination against COVID-19 started in the United Kingdom (UK) in December 2020 [1]. The short duration of protection from the primary course of COVID-19 vaccines and the emergence of COVID-19 variants of interest (e.g. coronavirus Beta variant) [2] meant booster doses were required to provide continued protection into Autumn 2021. In February 2021, the UK Department of Health and Social Care (DHSC) commissioned research to establish whether it was safe to receive the seasonal influenza and COVID-19 vaccines together. If both vaccines could be offered at the same appointment it was thought this would support vaccine uptake and reduce healthcare appointments.

Delivery of multicentre randomised controlled trials (RCTs) of investigational medicinal products typically takes years. The NIHR advise that RCT set-up (i.e. designing the trial, developing the protocol and securing permissions) can take between 6 to 12 months [3]. Site selection and opening is often staggered over several months to allow for staff training and local checks to take place. Recruitment often falls behind projected targets delaying the final analyses and publication of results [4].

The ComFluCOV trial was designed to be delivered, from conception to reporting of trial results, within 6 months to provide evidence to inform the 2021/2022 vaccination campaign. The main trial results have been reported previously [5]. In this report, we outline the key methodological design features and processes that allowed successful delivery of this RCT within accelerated timescales.

## Methods

### Trial inception

The trial was commissioned by the influenza policy team within the DHSC. The National Immunisation Schedule Evaluation Consortium (NISEC), who had already been commissioned to conduct other policy informing COVID-19 vaccine trials (COM-COV (ISRCTN 69254139); COV-Boost (ISRCTN 73765130); Preg-CoV (ISRCTN 15279830)), were approached through the Vaccine Task Force to consider this trial. A commissioned National Institute for Health Research (NIHR) - Policy Research Programme (PRP) funding call was open for approximately 1 week, which ComFluCOV was funded (NIHR203243, ISRCTN14391248).

### Trial design

ComFluCOV was a multicentre, parallel-group, placebo-controlled RCT designed to test the safety and immunogenicity of COVID-19 and seasonal influenza vaccine co-administration. The trial was designed in collaboration with the NISEC, the Vaccine Task Force and the Bristol Trials Centre (BTC), a UK Clinical Research Collaboration (UKCRC)-registered clinical trials unit (CTU) [6–8].

Several operational factors influenced the trial design: (1) the requirement to meet the reporting deadlines, set at 3 and 6 months from trial inception (March 2021); (2) the requirement to inform future policy for the co-administration of COVID-19 and influenza vaccines, whilst mass COVID-19 vaccination was still ongoing. The number of individuals who had received a first dose but not the second dose of COVID-19 vaccine was a key consideration in the trial design. COVID-19 booster doses were not due to take place for several months, therefore, a trial design using booster doses would not be feasible, so an alternative design was necessary; (3) availability of several COVID-19 and influenza vaccines in the vaccination programmes, meant data was needed to support as many of these products as possible. The trial was adapted to add another influenza vaccine after recruitment had commenced at the request of DHSC; and (4) the influenza vaccination season had concluded and the remaining influenza vaccine stock available for the trial was due to expire in Summer 2021. The influenza vaccine has a good safety profile, so whilst it was not intended to provide protection, an additional dose was considered low risk. Of the 679 participants (81%) 548 received their 2020/21 influenza vaccination prior to entering ComFluCOV.

**Fig. 1:**
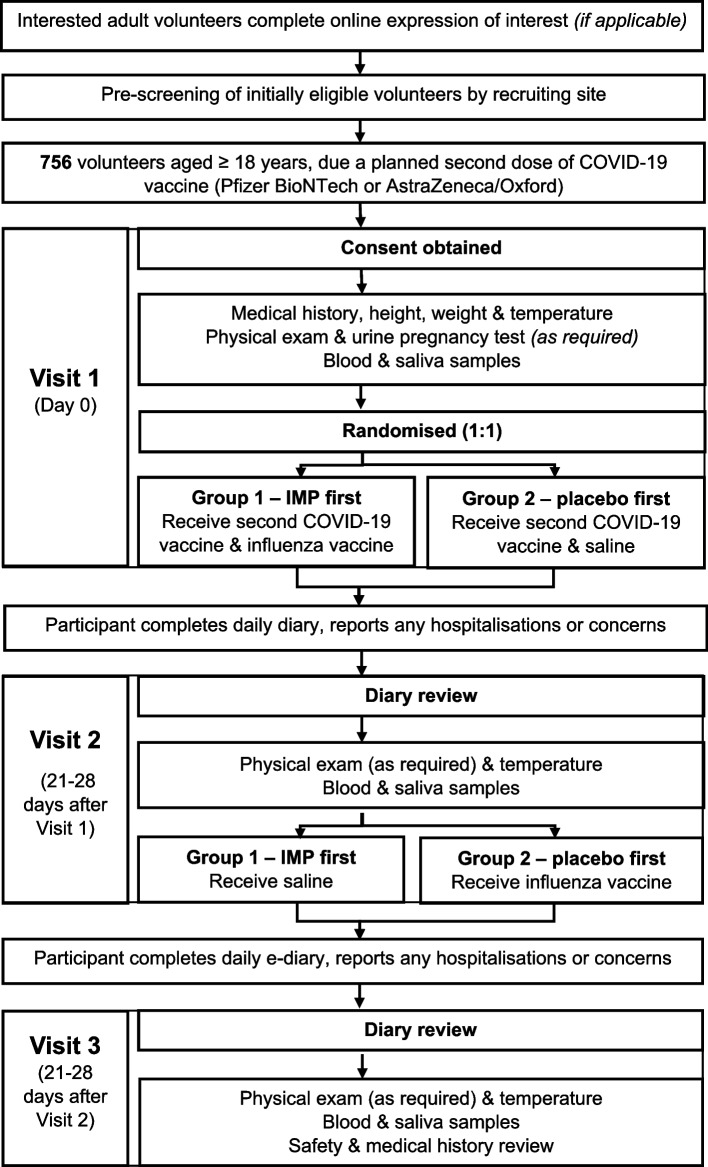
Trial schema outlining trial design

The trial evaluated two COVID-19 vaccines (ChAdOx1 or BNT162b2) and three seasonal, inactivated influenza vaccines (trivalent, MF59C adjuvanted or a cellular or recombinant quadrivalent vaccine). Volunteers aged 18 years and older, due to their second COVID-19 vaccine were eligible. Full inclusion and exclusion criteria are provided in the primary results publication [5]. Participants attended three visits, 3 weeks apart (see Fig. 1 for an overview of the trial design). All participants received their second COVID-19 vaccine at their first trial visit, co-administered with either an influenza or placebo vaccine. Placebo (saline) injections were used to maintain blinding. Participants, laboratory staff and clinicians assessing causality of adverse events were blinded to the treatment given. The bang blinding index [9] at visit 3 showed successful blinding. The primary outcome was obtained from participant-completed electronic diaries, completed for 7 days after each vaccination. The sample size was initially set at 504 participants (two COVID-19 vaccines and two influenza vaccines; four cohorts,) but was increased to 756 participants when the trial was adapted to include a third influenza vaccine (two additional cohorts).

### Trial management

The BTC were enlisted to deliver the trial with the co-investigators. Establishing an adequately sized, experienced trial team was key to expedite trial delivery. This was facilitated by redeployment of staff from other trials within the BTC to prioritise this nationally important trial.

### Regulatory approvals

Approvals from the Medicines and Healthcare products Regulatory Agency (MHRA), the Research Ethics Committee (REC) and the Health Research Authority (HRA) were expedited by obtaining Urgent Public Health status.

### Site recruitment and set up

In the 6 months preceding the ComFluCOV trial, the NIHR had provided infrastructure funding for trial sites willing to undertake COVID-19 vaccine trials, including regional vaccine delivery groups who were on standby to deliver Urgent Public Health vaccine trials. Initial site recruitment was performed using the West of England regional vaccine sites. Further site recruitment for the additional influenza vaccine was performed through the Clinical Research Network (CRN), focusing on sites that had received infrastructure funding, had the capacity to run the study, sufficient numbers of eligible participants in their local area (these data were facilitated by NHS England), and were using vaccines permitted by the protocol (for example, sites using Moderna vaccines were excluded).

Training of site staff was achieved remotely through online ‘site initiation visits’ (SIVs), provision of electronic training materials including videos and a trial manual. SIV training sessions were open to all sites, and not tailored for one specific site.

### Participant recruitment

National public interest in COVID-19 vaccine research facilitated promotion of the trial and encouraged volunteer engagement. Participants were recruited via advertising campaigns on the television, the radio, in newspapers, press and through social media. Volunteers were directed to a public website, developed by the BTC, similar to recruitment websites used in previous COVID-19 vaccine trials. The website included trial information and a questionnaire (developed by the Oxford Vaccine Group collaboration (part of Oxford Primary Care & Vaccines Collaborative UKCRC registered CTU) [10]) which volunteers would select their chosen site and complete initial trial screening inclusion and exclusion criteria questions. Only volunteers who met the initial inclusion criteria had their details passed onto the trial site. Potential participants were contacted by telephone for further screening assessment, and those potentially eligible were booked in for the first visit at their local site. At the first visit, a member of the trial team explained the trial, confirmed eligibility and received informed consent from those willing to take part in the trial. Some sites also made direct contact with people who were due to attend their second COVID-19 vaccine. Local CRNs provided support by contacting volunteers who had completed the questionnaire, and were able to do so outside of standard working hours, i.e. Bank Holidays.

### Trial data collection

Participants provided written informed consent on paper. Electronic consent was not deemed appropriate due to the short set-up timelines and use of volunteers not known to local teams. All other data capture was electronically, entered directly onto a secure purpose-designed password-protected trial database developed in REDCap (https://www.vanderbilt.edu/) by the BTC. A bespoke electronic trial management system (e-TMS) provided the following functions: (1) staff delegation log; (2) curriculum vitae (CV) and Good Clinical Practice (GCP) certificate upload facility and repository; (3) site staff training record; and (4) repository for approved trial documents (e.g. protocol, information sheets).

### Supply of investigational medicinal products

COVID-19 vaccine supply was maintained centrally by the UK government, it was accessed through the Vaccine Task Force and was supplied by Public Health England. Influenza vaccines were managed by the DHSC using contracts with individual suppliers. Stock was shipped directly to sites by the suppliers, or where this was not feasible by the lead pharmacy, University Hospitals Bristol and Weston NHS Foundation Trust (UHBW). Sites were advised to return excess COVID-19 vaccinations to the mass vaccination programmes. Excess expired influenza vaccines were destroyed.

### Laboratory analyses of tissue samples

Blood and saliva samples were collected from each participant at each trial visit. Sites were provided with instructions for the collection, processing and storage of samples within 24 h of collection. To reduce resource requirements, pre-printed, barcoded and human-readable sample labels for each sample type and visit were provided by the BTC team to each site. Sample inventories were drawn up from the data entered onto the trial database. The BTC arranged sample packing materials and couriers for all sites ensuring that sample aliquots were directly transported to the appropriate laboratory at the earliest opportunity.

The COVID-19 vaccine assays performed on the samples were relatively new. Two laboratories (Porton Down laboratories (Public Health England, UK) and Nexelis Laboratories Canada Inc.), used for previous COVID-19 vaccine trials, were chosen by the Vaccine Task Force for this trial. Allowing for increased consistency in analysis and possible comparisons between different COVID-19 vaccine trials.

### Monitoring and oversight

Central monitoring of protocol compliance, data quality, and participant safety followed a pre-specified plan agreed with the Sponsor, UHBW, and was signed off prior recruitment commencing. This included triggered site visits where poor data quality, protocol adherence concerns, or unusually low or high frequency of serious adverse event reports were identified, and could not be resolved remotely.

Study data was centrally checked by the BTC team daily to identify any missing or inconsistent entries. Any queries raised from checks were sent to sites on the same day that data were entered. To reduce the burden of staff manually checking patient-reported electronic diaries on a daily basis, delegated clinicians at site were sent daily extracts of electronic diaries, highlighting higher severity symptoms needing further information. Adverse events which met the protocol’s serious criteria underwent standard safety reporting procedures for clinical trials of investigational medicinal products in the UK [11–13]. Sites were also provided with checklists to support local compliance monitoring; for example, a dedicated local monitor, not responsible for consenting study participants, reviewed all consent forms in real time and completed a checklist which BTC reviewed centrally. Central monitoring took place 7 days a week, during recruitment. On clinic days, checks were performed after morning and afternoon clinics. Weekly online site meetings took place to keep site staff informed with progress and updates.

For efficiency, and on the advice of the DHSC, the Trial Steering Committee (TSC) and Data Monitoring and Safety Committee (DMSC) used to oversee ComFluCOV, had been previously established for similar COVID-19 trials, having knowledge about the topic and safety profile of the COVID-19 vaccines.

### Trial reporting

Trial analyses and reporting followed a pre-specified statistical analysis plan. All analyses were undertaken by two statisticians independently in parallel and the results compared. Details of the statistical input into the trial are reported separately.

## Results

### Trial inception

The BTC was chosen by NISEC to lead on ComFluCOV based on capacity and capability to deliver. Initial discussions commenced in late February 2021. Work started on 1st March 2021 with no funding in place or any guarantee it would be awarded. The protocol, trial documentation and regulatory applications were prepared and set up started in parallel with the development of the grant application. The DHSC confirmed the intent to fund the trial on 16th March 2021. The grant application, completed through collaboration with the BTC, Sponsor, Vaccine Task Force, DHSC and other NISEC investigators, was submitted on 26th March 2021. The formal contract was signed, on the 19th of May 2021.

### Trial design

The trial was designed to recruit volunteers due to receive their second dose of COVID-19 vaccine as a proxy for booster doses. Data supplied by NHS England showing that a large proportion of the population had already received their first dose of a COVID-19 vaccine, which made recruitment more feasible and results would be available for the start of the booster season. Cohorts were based on the two main COVID-19 vaccines used in the UK at the time, and the main influenza vaccines used for different age groups (under and over 65) (see Table 1).

**Table 1:** Participant recruitment numbers within each trial cohort

**Influenza vaccines**	**Recruitment numbers per cohort**	
	**COVID-19 vaccines**	
	ChAdOx1	BNT162b2
Trivalent, MF59C adjuvanted (65 years and over)	146	79
Cellular quadrivalent (under 65 years)	129	139
Recombinant quadrivalent (under 65 years)	128	58

Note: The recruitment target for each cohort was 126

Unlike most vaccine trials, blood was sampled 3 weeks after vaccination, instead of four, to reduce the trial duration whilst allowing sufficient time for the response to a secondary vaccine dose to be adequately detected. Meaning the interventional phase achieved complete participant datasets within just 6 weeks, (three visits 3 weeks apart, with vaccines administration at the first two).

The trial was blinded, but due to timelines using a matched placebo was not feasible, so saline injections were used. Participants were blinded by covering syringes with blinding tape and asking participants to look away during the injection. Site outcome assessors were blinded.

**Fig. 2:**
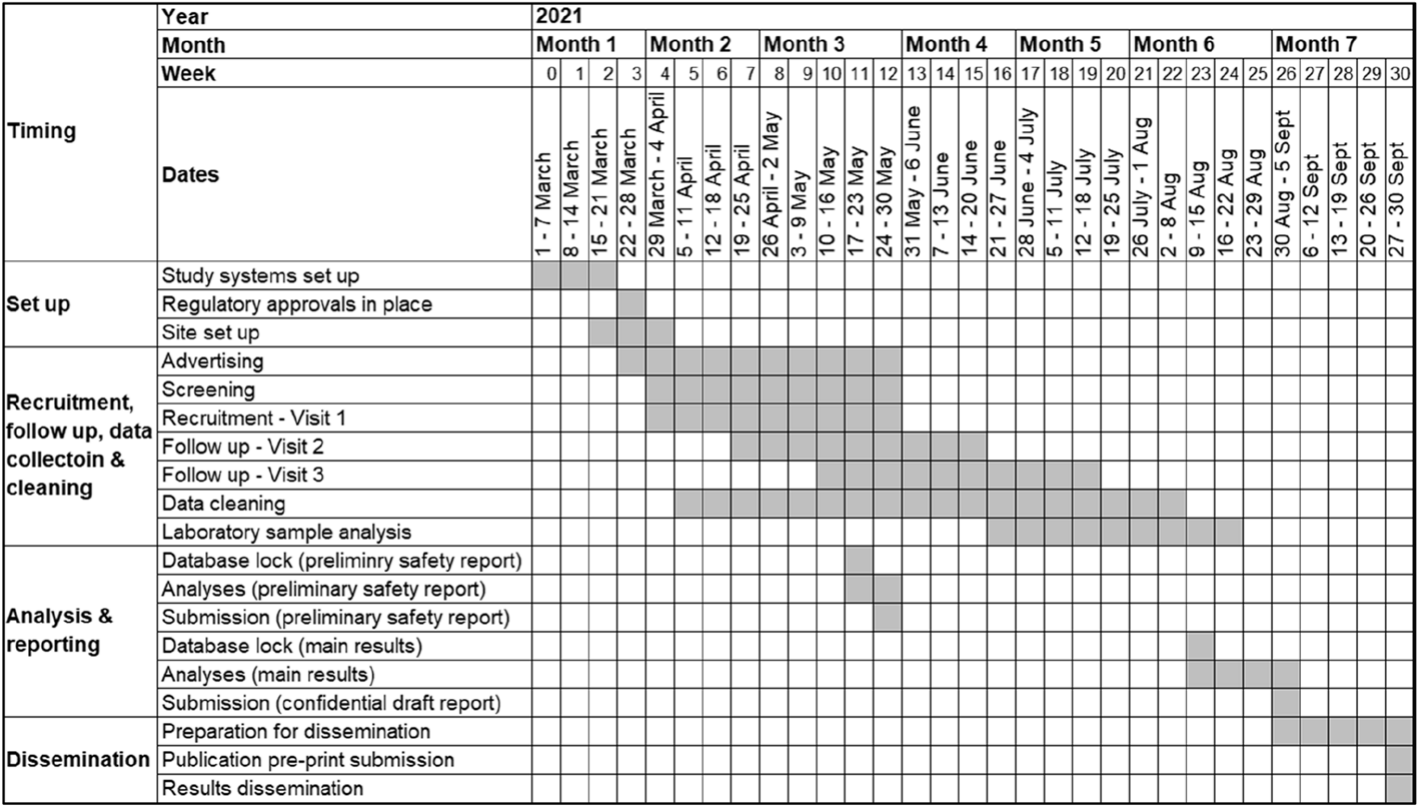
Gantt chart outlining the delivery timelines for the ComFluCOV study

To deliver the trial within the required timescales, it was necessary to (1) set up the trial within 1 month; (2) recruit and follow up all participants within three and a half months; (3) complete data cleaning and laboratory analyses within 1 month and, and (4) analyse and report the data to policy makers within 1 month, by September 2021, with a preliminary safety report in May 2021. Publication and dissemination had to be completed within a month, for national advertising campaigns. Figure 2 shows a Gantt chart outlining the trial timelines.

### Trial management

The BTC enabled immediate provision of an experienced central trial management team, consisting of five trial managers, two statisticians and three database managers and senior oversight provided by the BTC lead. These 11 individuals worked full time during the first four months, reducing to eight full time individuals in the last two months. These staff worked over 1600 h of overtime from March 2021 to August 2021.

To ensure rapid set up, individuals worked on multiple tasks in parallel which would usually be completed sequentially by a single person (see Table 2 for examples of trial set up tasks and the resource needed to complete them). A rota was used to manage workload allocating cover of daily tasks, for example pharmacovigilance checks, monitoring and being the point of contact for sites, 7 days a week between 7am and 10pm. Additional members of the wider BTC supported central monitoring and activities usually managed by sites, such as volunteer screening and localisation of trial documents.

**Table 2:** Examples of trial set up tasks completed within 1 month and the BTC resource required

**Task**	**BTC resource**										
	BTC lead	Trial manager 1	Trial manager 2	Trial manager 3	Trial manager 4	Trial manager 5	Statistician 1	Statistician 2	Database manager 1	Database manager 2	Database manager 3
Protocol development	✔	✔	✔								
Patient facing materials	✔		✔	✔	✔						
Advertising materials				✔							
Database development						✔	✔	✔	✔	✔	
Randomisation system						✔		✔	✔	✔	✔
Trial management system					✔						✔
Regulatory approvals		✔									
Sponsorship/governance			✔								
Training documents		✔	✔	✔	✔	✔					
Pharmacy			✔								
Laboratory/samples		✔									
Monitoring plan			✔								
Site set up					✔						
Statistical analysis plan	✔						✔	✔			

Note: All set up tasks were overseen by the CI and BTC lead. All BTC staff were full time employees

Trial processes were expedited to meet the deadlines. The protocol was finalised within 2 weeks by the Chief Investigator and BTC lead with input from the co-applicants, BTC team and review by the Sponsor. Participant-facing documentation was prepared in parallel within 3 weeks by three trial managers and the BTC lead, utilising existing BTC templates and example documentation provided by the Oxford Vaccine Group collaboration. Due to time constraints it was not possible for a Patient and Public Involvement (PPI) group to review participant-facing documents. However, they were closely based on templates previously reviewed by PPI. Localisation of documents with site details was completed by the BTC team ahead of site greenlight to minimise delays. During set-up, the Sponsor allowed parallel completion of tasks, normally undertaken sequentially, and permitted set-up activity, such a completing the risk assessment to be undertaken before funds were secured and contracts were in place, and commencing the data management plan whilst the protocol was being drafted.

The BTC team held weekly online meetings with all the stakeholders, the Sponsor and sites to keep everyone informed of progress and developments and daily internal meetings during the set-up period. The CI and a BTC representative joined weekly Vaccine Task Force and NISEC meetings to present trial progress. Due to the time constraints it was not feasible to include a public member as part of the trial management team.

### Regulatory approvals

In March 2021, the NIHR classed ComFluCOV as an Urgent Public Health trial to be prioritised by the Vaccine Task Force. Urgent Public Health status facilitated access to expedited regulatory approvals and direct lines of communication with the REC, HRA and MHRA who reviewed documents and provided feedback ahead of formal regulatory submissions. A dedicated Sponsor team worked on expediting trial governance processes. The trial was allowed to bypass the REC booking system and the REC provided a review slot in a meeting that was already full. The REC, South-Central Berkshire REC (21/SC/0100) chosen for their previous involvement in related COVID-19 vaccine RCTs, received the trial documents 4 days prior to the meeting instead of the usual 3 weeks. Approval was received from the REC 6 days after submission of the initial REC application, following a minor amendment, requested and addressed on the fifth day. The HRA provided a letter of ‘partial’ approval allowing trial advertising, screening and recruiting activities to take place ahead of MHRA approval. MHRA approval (EudraCT 2021-001124-18) was received 8 days after the MHRA submission. The HRA issued ‘full’ approval on the same day that MHRA approval was received, 1 day after the ‘partial’ HRA approval. Had the MHRA taken longer to approve the trial, participants could still have been recruited and been ready for trial interventions to begin once all the regulatory approvals were in place.

### Site recruitment and set-up

Sites prioritised set-up and delivery of Urgent Public Health badged trials, which was key to rapid opening. Site set-up took place in parallel to local capacity and capability reviews and contract sign-off. The first six sites set up were NHS hospital trusts, within the West of England regional vaccine delivery group, experienced in delivering COVID-19 research trials with similar trial and data collection processes. Overall, trial set-up was achieved within 1 month.

The first SIV took place at the end of March 2021, prior to full HRA and MHRA approval. Staff from the first six sites attended, and knowledge sharing between sites was encouraged. The additional influenza vaccine meant a further six sites, three general practice (GP) surgeries, a COVID-19 mass vaccination centre and two NHS hospital trusts, unfamiliar with COVID-19 vaccine research, were brought on board to support the additional recruitment. Three more SIVs occurred between the end of April 2021 and early May 2021. Staff unable to attend an SIV were able to receive local training, access recordings of the SIV and other trial training materials.

Twelve sites in total were set up across England and Wales, with over 380 site staff trained remotely within two months. In addition, local CRNs screened and booked in potentially eligible participants for their first trial visit. The time between SIVs, site greenlight and the first trial visit is summarised in Table 3. Sites took on average 2 weeks to recruit their first participant following the SIV, with no site taking longer than 1 month.

**Table 3:** Timing of site initiation visits (SIVs), greenlight and first clinics for each site

**Site**	**Date of SIV**	**Number of days between SIV and greenlight**	**Number of days between greenlight and date of first clinic (visit 1)**
NHS Hospital Trust 1	24/03/21	7	1
NHS Hospital Trust 2	24/03/21	15	2
NHS Hospital Trust 3	24/03/21	15	2
NHS Hospital Trust 4	24/03/21	26	1
NHS Hospital Trust 5	24/03/21	15	8
NHS Hospital Trust 6	24/03/21	16	1
Mass Vaccination Centre 1	26/04/21	14	4
NHS Hospital Trust 7	04/05/21	8	5
NHS Hospital Trust 8	07/05/21	4	6
General Practice 1	04/05/21	6	7
General Practice 2	07/05/21	4	2
General Practice 3	07/05/21	5	3

### Participant recruitment

An extensive advertising campaign facilitated by the Sponsor communications team went live 2 days after full HRA approval, on Friday 26^th^ March 2021. The campaign included a press release, email communications, social media, television, radio and newspaper and resulted in over 30 pieces of coverage. Over 340 volunteers registered their interest, via the online questionnaire, over the first post-advertising weekend and interest continued to increase through the recruitment period totalling 3535 registrations. Telephone screening of volunteers commenced 3 days after the press release, on Monday 29^th^ March 2021. The Urgent Public Health status enabled trial sites priority access to CRN support, which alongside BTC staff, assisted sites by completing screening phone calls throughout recruitment, and booked over 100 potentially eligible participants for their first trial visit in the first week of recruitment.

**Fig. 3:**
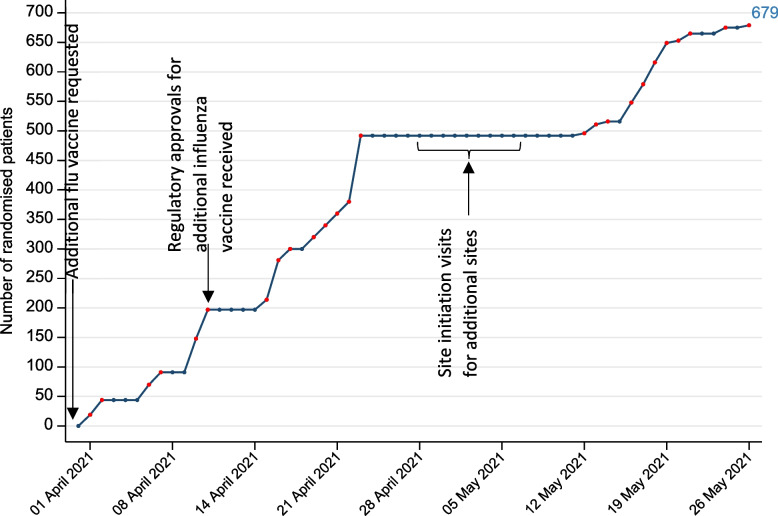
Participant recruitment over time

Larger sites (hospitals and a mass vaccination centre) arranged specific clinics for each trial visit; GP practices conducted trial activities alongside day-to-day clinical activity. Recruitment commenced on 1^st^ April 2021. Sites recruited to their maximum capacity, with full clinics of between 20 to 50 participants. A total of 679 (90%) of the target 756 participants were recruited in two months (see Fig. 3). The stepped recruitment graph reflects *en masse* participant recruitment at clinics. Recruitment dropped towards the end of April and in early May as additional sites were set up following the incorporation of the third influenza vaccine. Participant follow-up rates were high with less than 2% not attending follow-up visits; 671/679 participants attended visit 2 and 670/679 participants attended visit 3. The final trial visit was held in July 2021. Reasons participants were not recruited are reported in [5]. However, with additional resource, allowing sites to open faster and offer more clinic dates it may have been possible to recruit participants more rapidly.

### Trial data collection

A bespoke e-TMS, based on e-TMSs previously developed for BTC trials, was developed by a database manager and a trial manager within 2 weeks. It provided a simple and efficient way for over 380 site staff to electronically register for the trial and Principal Investigators to remotely review staff prior to approving them to work on the trial. The central coordination team could provide the correct level of access to the patient database, without requiring wet ink signatures or transfer of paper/email documents. Two database managers and a trial manager developed the participant database, within 3 weeks, by adapting data specifications from other COVID-19 vaccine trials provided by the Oxford Vaccine Group collaboration. Using a simple layout, guiding the completion of the clinic visits, and a format which sites were already familiar with, reduced the time required for staff training. Data collection was kept to a minimum, focusing on data to confirm eligibility, study outcomes and protocol adherence, making data collection light-touch for sites. All data capture was electronic and entered directly onto the participant database whilst in clinic, allowing for efficient, paper-light delivery at sites. The Oxford Vaccine Group developed the questionnaire for volunteers to register interest in taking part in the trial within 3 weeks. All systems and databases were tested and signed off by the BTC prior to go-live.

### Supply of investigational medicinal products

Two influenza vaccines were included from the start; one was appropriate for people aged 65 years and over and the other was appropriate for people under 65 years (see Table 1). Following the addition of a third influenza vaccine which was also appropriate for the under 65 years age group, it was agreed that sites would only receive stock of one particular age-appropriate influenza vaccine for participants under 65 years of age to minimise vaccination errors. No site recruited to all six trial cohorts. All trial sites promptly received the correct IMP stock preventing delay to site opening.

### Laboratory analyses of tissue samples

BTC staff closely monitored data on the processing and storage of samples and requested resolution of any queries within 24 h of sample collection. Providing pre-printed human readable and barcoded sample labels containing the study ID, visit and aliquot number expedited the labelling process, prevented transcription errors and helped sites identify samples and resolve sample queries promptly. Samples were stored in pre-packed cryoboxes containing everything needed for each shipment, to minimise the time needed to prepare samples for transportation. The barcode on the sample labels was critical for efficient processing of samples by the laboratories, since they could just scan the barcode and did not have to decipher handwritten study ID and visit numbers. Upon completion of all second trial visits in early July 2021, serum samples from trial visits 1 and 2 were sent to Nexelis laboratories, Canada, to assess the immune response to a second dose of COVID-19 vaccine. Upon completion of all third trial visits in July 2023, serum samples from trial visits 1, 2 and 3 were sent to Porton Down laboratories, UK, to assess whether a response to influenza vaccine had been generated. The remaining trial samples were shipped from sites to the BTC to be held in UHBW / University of Bristol laboratories. Samples where participants had consented to further analyses, were transferred to the Bristol biobank and those without consent for further analyses were destroyed.

Following receipt of the samples, the Nexelis laboratory informed the BTC team that they would be unable to process the samples within the timelines required by the trial, due to the high volume of samples to analyse for competing COVID-19 trials. Porton Down laboratories were able to complete a serology assay equivalent to the Nexelis assay to assess response to the second dose of COVID-19 vaccine from which results would be available within the required timelines. As a result, additional serum samples from trial visits 1 and 2 were sent to Porton Down laboratories in late July 2021 for this assay to be completed.

Laboratory results received from Porton Down both for the immune response to COVID-19 and influenza vaccines in August 2021 were included in the main publication in September 2021 [5]. Laboratory results received from Nexelis in October 2021, were not included in the main publication, however, were shared with the DHSC in December 2021 and will be published. In addition, two secondary outcomes; 1) Investigation of mucosal immune responses to COVID-19 vaccines in saliva by the University of Bristol laboratories; and 2) Neutralising antibodies against SARS-CoV-2 measured in serum to assess response to second dose of COVID-19 vaccine measured by Porton Down, were not reported in the main publication following delayed sample analyses due to lack of capacity and prioritisation of other competing trials. These analyses have now been completed and will be published.

### Monitoring and oversight

Daily data cleaning using database reports and checklists took place throughout the recruitment and follow-up period. Data queries were resolved primarily through email communications, weekly site meetings and occasionally phone calls with sites. In addition to daily monitoring queries, additional and unresolved queries were sent to sites at least once every 2 weeks until resolved. Queries sent via email were logged on a spreadsheet. Unresolved queries were checked periodically and chased through to resolution, with emails saved for transparency. Sites generally worked with the BTC team to resolve issues rapidly. Any issues identified with checklists completed by site-based monitors, were returned to the site the day they were completed to address in real time. All database queries were resolved, and the participant database was locked in August 2021, 6 weeks after the final participant follow-up. No triggered onsite monitoring was required.

The TSC and DMSC committees were set up quickly. Three reports were produced for the independent oversight committees outlining trial progress throughout the delivery period; April 2021, June 2021, and September 2021. The DMSC recommended continuation of the trial to completion and congratulated the trial team on recruitment, data quality and follow-up.

### Analyses and reporting

The statistical analysis plan was signed off in May 2021, ahead of formal analyses. A preliminary safety report describing the primary outcome results in the four original cohorts of participants following trial visit 1 was produced and sent to the DHSC in May 2021 to inform policy. This interim analysis was performed on validated data, 7 days after 473 participants had completed the primary safety outcome.

Analyses of all cohorts took place through August and September 2021. The final report outlining the main trial findings was sent to the DHSC, MHRA and JCVI in early September 2021. The main trial results were made publicly available during a press briefing at the end of September and via pre-print on the Lancet website. Due to the high-profile nature of the results, and to avoid any potential information leaks, an established BTC PPI group, who understood the need for confidentiality, reviewed the participant results leaflet. Feedback was requested within 24 h, responses from five out of eight members were received. Overall, the feedback was positive, and all PPI suggestions were incorporated to improve the leaflet. The dissemination of results to participants occurred on the same day as the press release by email via the database, when the results were made publicly available. The results were widely publicised with over 300 pieces of coverage about ComFluCOV online, in print and broadcasted within the first week of the results being made public.

The MHRA and JCVI published statements that based on the data provided it was safe to give COVID-19 booster doses at the same time as influenza vaccines [14]. These results also received international recognition and informed the World Health Organisation guidelines on co-vaccination [15, 16]. The trial results were submitted in September 2021 to the Lancet, who agreed a ‘fast-track’ journal review, and it was accepted for publication within 2 weeks of submission subject to addressing the reviewers’ comments and published in full in November 2021 [5].

## Discussion

It was possible to rapidly deliver a vaccine trial in the exceptional circumstances brought about by the COVID-19 pandemic. ComFluCOV reported the primary and key secondary outcomes within 6 months from the first conversation to publication. The trial milestones to inform the UK and World Health Organisation immunisation policy were met, showing it is safe to receive the influenza vaccine at the same time as a COVID-19 vaccination. The results were widely publicised and implementation of this practice is underway where feasible in the UK and internationally. Delivery of ComFluCOV within accelerated timescales was possible due to several key factors: (1) access to expedited approvals; (2) prioritisation of Urgent Public Health trials; (3) willingness of volunteers to partake in the trial; (4) significant human resource dedicated to delivering the trial; (5) collaboration and sharing of resources between CTUs; (6) development and use of databases and information technology (IT) systems. Some factors were as result of a unique set of circumstances created by the COVID-19 pandemic whilst others could be adopted outside of a pandemic as ‘business as usual’.

The following factors were critical to delivery of ComFluCOV but would be difficult to replicate outside of a pandemic situation. Access to expedited regulatory reviews was key to minimising the set-up period, including the facilitation of discussions and feedback ahead of regulatory submissions and applications being turned around within days rather than months. Whilst approvals were expedited, no processes were bypassed, ensuring regulatory compliance. Urgent Public Health trials were prioritised whilst other research was on put hold. This meant that regulators, CTUs, CRNs and sites were able to allocate all their available resource to deliver trials like ComFluCOV. Sites mobilised quickly, fast-tracking local trial set up and instead of a staggered start to recruitment, they ran dedicated clinics to allow efficient recruitment of large numbers of volunteers (up to 50 per clinic in ComFluCOV) in short periods of time. The vast number of people at risk of infection from COVID-19 meant that the pool of potentially eligible participants was very large. However, due to the timing of the trial this population reduced throughout the trial timeline as more people were vaccinated against COVID-19. As a result of the immense media publicity around COVID-19, large numbers of people were keen to take part in COVID-19 research during the pandemic, and this combined with the trial specific publicity, was reflected in the number of volunteers registering interest and consenting to participate in ComFluCOV. Staff dedicated many hours beyond their usual working patterns for the COVID-19 cause and both BTC and site staff changed their working patterns to ensure that there was cover for ComFluCOV throughout, whilst others covered other ongoing work.

Some of the elements of the trial which could be adopted to increase efficiency of future trials are outlined below. Working with a CTU infrastructure enabled immediate mobilisation of a team of experienced researchers. This mobilisation took place before trial funding was secured, which was a risk to both the Sponsor and BTC, but the risk was considered acceptable as the trial had been requested by DHSC. Knowledge of working practices and having established ways of working amongst the BTC staff simplified team working and made it possible to split work and carry out tasks in parallel. Collaboration and sharing of resources between CTUs enabled efficient development of trial materials and data collection tools. For example, Oxford Vaccine Group provided BTC with trial materials from previous COVID-19 vaccine trials of similar design, including participant-facing documentation, and database specifications, which enabled rapid development of the documentation and systems. It also meant that some COVID-19 vaccine research sites were familiar with the systems and processes used. The collaboration with the Oxford Vaccine Group and NISEC helped make the most of learning from other similar trials and issues at a national level, for example, anticipating issues with capacity for sample analyses at previously used laboratories and finding alternative laboratories which could do equivalent work. The use of IT systems can be critical in enabling efficient delivery of trials. In ComFluCOV, the use of electronic trial materials, made delivery at site efficient and paper light and allowed for real-time data entry and central monitoring, which in turn meant that any problems were identified as they happened. The use of an eTMS, electronic delegation logs and SharePoint enabled efficient remote working, as multiple members of staff could access shared resources. Implementation of short, regular, online meetings and drop-in sessions through Microsoft Teams was essential to communicate with stakeholders keeping everyone informed of progress and upcoming changes. The Chief Investigator for ComFluCOV and BTC are undertaking another co-vaccination trial which will utilise much of the learning, systems and processes used for ComFluCOV.

## Limitations

As a result of the national vaccine rollout guidelines, the pool of eligible volunteers diminished quickly with time and as a result the target sample size of 756 participants was not reached. Although the trial included the first two licenced COVID-19 vaccines in use at the inception of the trial, and included the influenza vaccines used most frequently in UK vaccination programmes, it was not possible to include all combinations of COVID-19 and influenza vaccines. Another limiting factor was the time pressures resulting from influenza vaccine expiry dates. Due to the short timescale to identify potential sites and complete full feasibility checks, some sites were recruited but they did not have all the necessary research infrastructure in place (e.g. limited access of GP practices to -80 degree freezers, mass vaccination centres not having weighing scales, smaller sites having limited number of research staff). Some sites did not have sufficient potentially eligible people to recruit the volume of participants required within the short timescales, resulting in under-recruitment to certain cohorts. As described in the results section, not all outcomes were reported in the primary results paper. Whilst successful delivery of ComFluCOV was achieved, other work had to be paused to achieve it.

## Conclusions

ComFluCOV was delivered within 6 months from conception to delivery of the main results. Delivering this multicentre RCT within accelerated timescales was possible in part due to a unique set of circumstances created by the pandemic situation. However, this is not reproducible under normal circumstances and with usual resourcing. Lessons learnt such as the use of IT systems and collaborations should be adopted for efficient delivery of future trials outside of a pandemic situation.

## Data Availability

Not applicable as methodology publication

## Abbreviations

BTC: Bristol Trials Centre
BNT162b2: COVID-19 mRNA Pfizer vaccine
ChAdOx1: COVID-19 AstraZeneca vaccine
CTU: Clinical trials unit
CRN: Clinical Research Network
CV: Curriculum vitae
DHSC: Department of Health and Social Care
DMSC: Data Monitoring and Safety Committee
e-TMS: Electronic-Trial Management System
GCP: Good Clinical Practice
GP: General practice
HRA: Health Research Authority
JCVI: Joint Committee on Vaccination and Immunisation
MHRA: Medicines and Healthcare Regulatory Agency
NIHR: National Institute for Health Research
NISEC: National Immunisation Schedule Evaluation Consortium
OVG: Oxford Vaccine Group
PHE: Public Health England
PPI: Patient and Public Involvement
PRP: Policy Research Programme
RCT: Randomised controlled trial
REC: Research Ethics Committee
SIV: Site initiation visit
TSC: Trial Steering Committee
UHBW: University Hospitals Bristol and Weston NHS Foundation Trust
UKCRC: Clinical Research Collaboration
UPH: Urgent Public Health
VTF: Vaccine Task Force
